# Prevalence of comorbidities associated with sickle cell disease among non-elderly individuals with commercial insurance–A retrospective cohort study

**DOI:** 10.1371/journal.pone.0278137

**Published:** 2022-11-29

**Authors:** Scott D. Ramsey, M. A. Bender, Li Li, Kate M. Johnson, Boshen Jiao, Beth Devine, Anirban Basu

**Affiliations:** 1 Division of Public Health Sciences and Hutchinson Institute for Cancer Outcomes Research, Fred Hutchinson Cancer Research Center, Seattle, WA, United States of America; 2 The Comparative Health Outcomes, Policy & Economics (CHOICE) Institute, Department of Pharmacy, University of Washington, Seattle, WA, United States of America; 3 Department of Pediatrics, University of Washington, Seattle, WA, United States of America; 4 Clinical Research Division, Fred Hutchinson Cancer Research Center, Seattle, WA, United States of America; 5 Department of Pharmaceutical Sciences, University of British Columbia, Vancouver, Canada; 6 Harvard T.H. Chan School of Public Health, Harvard University, Boston, MA, United States of America; 7 Department of Health Services, University of Washington, Seattle, WA, United States of America; 8 Department of Biomedical Informatics and Medical Education, University of Washington, Seattle, WA, United States of America; 9 Department of Economics, University of Washington, Seattle, WA, United States of America; University of Illinois at Chicago, UNITED STATES

## Abstract

Sickle cell disease (SCD) is a severe monogenic disease associated with high morbidity and mortality and a disproportionate burden on Black communities. Few population-based studies have examined the prevalence of comorbidities among persons with SCD. We estimated the prevalence of comorbidities experienced by individuals with SCD enrolled in employer-based health insurance plans in the US over their non-elderly lifetimes (0–64 years of age) with a retrospective cohort design using Truven Health MarketScan commercial claims data from 2007–2018. ICD-9/10 codes were used to identify individuals with SCD using a previously published algorithm. For this cohort, comorbidities associated with SCD were identified across 3 age categories (<18, 18–45, 46–64 years-old), based on the CMS Chronic Comorbidities Warehouse or SCD-specific diagnosis codes, when applicable. The total number of SCD patients available for analysis in each age category was 7,502 (<18 years), 10,183 (18–45 years) and 4,459 (46–64 years). Across all ages, vaso-occlusive pain, infections (non-specific), and fever were the most common comorbidities. Vaso-occlusive pain and infection were the most prevalent conditions for persons age <18- and 18–45-year-olds, while in the 46–54-year-old age group, infection and cardiovascular including pulmonary hypertension were most prevalent. Compared to persons <18 years old, the prevalence of vaso-occlusive pain, fever, and acute chest syndrome claims declined in older populations. The comorbidity burden of SCD is significant across all age groups. SCD patients experience comorbidities of age such as chronic pain, cardio-vascular conditions including pulmonary hypertension and renal disease at far higher rates than the general population. Novel disease modifying therapies in development have the potential to significantly reduce the comorbidity burden of SCD.

## Introduction

Sickle-cell disease (SCD), an inherited blood disorder caused by a mutation in the β-globin gene, affects approximately 100,000 individuals in the US, primarily of African-American descent, and is associated with lifelong morbidity and increased risk of mortality [1]. While acute vaso-occlusive pain episodes are the hallmark of SCD, individuals with this condition experience numerous other debilitating and life-threatening comorbidities throughout their lifetimes [2–4]. Despite the widespread recognition that comorbidities are a major contributor to the burden of SCD, population-based prevalence estimates of comorbidities are uncommon and typically limited to specific comorbidities, institutions, or registries. A more comprehensive understanding of the prevalence of common comorbidities for SCD patients in large populations is important at this time. A number of very costly but potentially curative therapies for SCD are on the horizon [5], with ten gene therapies currently in the clinical testing stage [6]. These therapies have the potential to revolutionize care for SCD by substantially reducing or eliminating the most debilitating comorbidities that occur over time in this population. However, with expected prices of nearly $2 million [7, 8] per treatment, issues of affordability and access will be points of contention between patients, payers, and policy makers [9, 10]. An important component of estimating the potential impact of gene therapies on the burden of SCD is gaining a better understanding of the current burden of SCD-related comorbidities in large, representative populations. Accordingly, the purpose of this study was to estimate the prevalence of common comorbidities, by age group, in a national population of commercially insured individuals with SCD.

## Methods

### Data sources

We conducted a retrospective cohort study using a cohort of individuals with SCD using the Truven Health MarketScan Commercial Claims and Encounters Database. The Truven database contains claims data for over 115 million individuals and their dependents from all 50 states with employer-sponsored private health insurance. These data include outpatient and inpatient medical claims, prescription drug claims, health utilization records, payer and individual costs, and demographic characteristics of individuals, including their age at enrollment, sex, geographic region, and insurance plan type. Race is not available in the data. The approving Institutional Review Board (IRB) for this study is the Fred Hutch Institutional Review Board. The need for consent was waived by the IRB based on the study’s classification as secondary research; the identifiable private information used in the study is both publicly available and de-identified.

### Study population

We created a retrospective longitudinal cohort of individuals from the Truven database who fulfilled a previously validated case definition for SCD at any point between 2007 and 2018 [11]. To be included, individuals had at least one inpatient claim or two outpatient or emergency department (ED) claims for SCD in any position using International Classification of Disease, 9th and 10th edition codes (ICD-9 282.6, 282.41, 282.42, ICD-10 D57, D57.8, excluding 282.5 and D57.3; sickle cell trait). This approach is based on an algorithm that has demonstrated over 90% sensitivity and specificity in identifying individuals with SCD [11]. Individuals were followed from the start of their first claim encounter (irrespective of when SCD was diagnosed) beginning from January 1, 2007 until December 31, 2018. Using date of birth, we calculated patient’s ages at each month of follow-up, so that we could record age at the initial appearance of the complication of interest. SCD patients with unknown age were excluded from the analysis. We also excluded persons with records starting on or after age 65 and observations of individuals for periods with ages ≥65 years to eliminate periods in which they were eligible for Medicare.

### Identifying comorbidities related to SCD

Individual comorbidities associated with SCD were compiled from the literature and reference texts, with review by the study team. To identify comorbidities, we searched inpatient, ambulatory, and ED claims records of the study population for ICD-9/10 codes indicating those comorbidities. When possible, we used claims algorithms developed by the Centers for Medicare and Medicaid Services (CMS) for its Chronic Comorbidities Warehouse (e.g., stroke, asthma) [12]. For comorbidities that are more specific to SCD, we used algorithms previously published in the literature or, when necessary, searched ICD files for relevant codes. Appendix A contains the complete list of comorbidities and ICD codes used for this study.

Using the date of the initial claim for each condition, we calculated prevalence for each comorbidity by age group–less than 18, 18 to 45, 46 to 64. To increase the likelihood that the initial claim accurately reflected the first time of occurrence of the condition, we required patients to have at least 12 months of continuous enrollment prior to the date of first occurrence in each age window. We removed this restriction in a secondary analysis.

To account for the fact that some comorbidities may resolve or not reoccur in later age groups (e.g., asthma in children), the comorbidity algorithm was repeated for persons who “aged in” to an older age category.

## Results

We identified 20,206 unique patients who met eligibility criteria over the observation period. The median age in years at the time of the initial record was 24.0 (mean 25.7, SD 17.5). The median length of claims records for individual patients was 39 months (mean 50.1, SD 33.9). 42.7% were male and 57.3% were female (Table 1). The total number of SCD patients available for analysis in each age category was 7,502 (<18 years), 10,183 (18–64 years) and 4,459 (46–64 years).

**Table 1:** Demographic table by age group.

Group	<18	18–45	46–64	Total
**Total**	7,502	10,183	4,459	20,206
**Gender**				
Female	3,752 (50.01%)	6,155 (60.44%)	2,793 (62.64%)	11,574 (57.28%)
Male	3,750 (49.99%)	4,028 (39.56%)	1,666 (37.36%)	8,632 (42.72%)
**Region**				
Northeast	1,577 (21.02%)	1,985 (19.49%)	936 (20.99%)	4,119 (20.39%)
North Central	1,152 (15.36%)	1,522 (14.95%)	722 (16.19%)	3,089 (15.29%)
South	4,293 (57.22%)	5,875 (57.69%)	2,363 (52.99%)	11,438 (56.61%)
West	480 (6.40%)	801 (7.87%)	438 (9.82%)	1,560 (7.72%)

Table 2 shows the prevalence rates by age group with the 12-month enrollment requirement. Table 3 shows the prevalence of SCD–related comorbidities by service code–inpatient services, ER services, ambulatory services, and combined services. Table 4 shows prevalence rates without the 12-month enrollment requirement.

**Table 2:** Prevalence of SCD comorbidities by age group (12-month enrollment requirement).

Prevalence by Age Category**				
	N (%)			
Comorbidities*	<18	18–45	46–64	Total N
**Total**	** 7,502**	**10,183**	**4,459**	**20,206**
Vaso-occlusive pain	4435 (59.12%)	5728 (56.25%)	1773 (39.76%)	11310 (55.97%)
Infections (non-specific)	4270 (56.92%)	5231 (51.37%)	2335 (52.37%)	11369 (56.27%)
Fever	3784 (50.44%)	2635 (25.88%)	850 (19.06%)	7093 (35.10%)
Acute chest syndrome	2203 (29.37%)	2636 (25.89%)	960 (21.53%)	5641 (27.92%)
Cardiovascular including pulmonary hypertension	709 (9.45%)	2830 (27.79%)	2068 (46.38%)	5455 (27.00%)
Fatigue	481 (6.41%)	2446 (24.02%)	1644 (36.87%)	4433 (21.94%)
Asthma	1986 (26.47%)	1283 (12.6%)	656 (14.71%)	3750 (18.56%)
Chronic lung disease	1160 (15.46%)	1517 (14.9%)	1060 (23.77%)	3645 (18.04%)
Sleep disordered breathing and nocturnal hypoxemia	921 (12.28%)	1229 (12.07%)	753 (16.89%)	2826 (13.99%)
Chronic renal disease	320 (4.27%)	1279 (12.56%)	1250 (28.03%)	2778 (13.75%)
Hepatic and hepatobiliary complications	654 (8.72%)	1350 (13.26%)	710 (15.92%)	2670 (13.21%)
Chronic mental health disorders	282 (3.76%)	1341 (13.17%)	734 (16.46%)	2285 (11.31%)
Chronic pain	207 (2.76%)	1354 (13.3%)	723 (16.21%)	2216 (10.97%)
Stroke	462 (6.16%)	622 (6.11%)	696 (15.61%)	1727 (8.55%)
Bacteremia and sepsis	429 (5.72%)	915 (8.99%)	430 (9.64%)	1756 (8.69%)
Avascular necrosis and bone damage	233 (3.11%)	969 (9.52%)	378 (8.48%)	1517 (7.51%)
Splenic disease	680 (9.06%)	499 (4.9%)	238 (5.34%)	1389 (6.87%)
Acute renal failure	112 (1.49%)	563 (5.53%)	691 (15.5%)	1346 (6.66%)
Ocular complications	143 (1.91%)	522 (5.13%)	371 (8.32%)	1007 (4.98%)
Transfusion complications	356 (4.75%)	429 (4.21%)	123 (2.76%)	881 (4.36%)
Cognitive impairment	357 (4.76%)	176 (1.73%)	189 (4.24%)	711 (3.52%)
Hydroxyurea - thrombocytopenia	161 (2.15%)	255 (2.5%)	160 (3.59%)	571 (2.83%)
Hydroxyurea - leukopenia	142 (1.89%)	132 (1.3%)	133 (2.98%)	403 (1.99%)
Dactylitis	105 (1.4%)	160 (1.57%)	121 (2.71%)	386 (1.91%)
Priapism	95 (1.27%)	210 (2.06%)	18 (0.4%)	311 (1.54%)
Myocardial infarction	4 (0.05%)	91 (0.89%)	192 (4.31%)	284 (1.41%)
Multi-organ failure	21 (0.28%)	77 (0.76%)	70 (1.57%)	168 (0.83%)
Leg ulcers	2 (0.03%)	65 (0.64%)	48 (1.08%)	115 (0.57%)
Hydroxyurea—oligospermia/azospermia	0 (0%)	17 (0.17%)	3 (0.07%)	19 (0.09%)

*Combined = Inpatient (any position) or ER or ambulatory

**Prevalence in each age window. Each patient may be in multiple age windows

**Table 3:** Prevalence of SCD comorbidities by service code.

Prevalence by Service Code				
Comorbidities	Inpatient services	ER services	Ambulatory services	Combined*
Infections (non-specific)	4819 (23.61%)	3523 (17.26%)	9810 (48.06%)	11707 (57.36%)
Vaso-occlusive pain	7919 (38.8%)	7187 (35.21%)	9269 (45.41%)	11706 (57.35%)
Fever	4316 (21.15%)	3236 (15.85%)	4744 (23.24%)	7330 (35.91%)
Acute chest syndrome	4526 (22.17%)	1315 (6.44%)	3270 (16.02%)	5859 (28.71%)
Cardiovascular including pulmonary hypertension	2788 (13.66%)	1326 (6.5%)	4187 (20.51%)	5666 (27.76%)
Fatigue	653 (3.2%)	651 (3.19%)	4161 (20.39%)	4621 (22.64%)
Asthma	1332 (6.53%)	1059 (5.19%)	3450 (16.9%)	3868 (18.95%)
Chronic lung disease	1915 (9.38%)	432 (2.12%)	2451 (12.01%)	3746 (18.35%)
Sleep disordered breathing and nocturnal hypoxemia	1651 (8.09%)	296 (1.45%)	1774 (8.69%)	2919 (14.3%)
Chronic renal disease	1459 (7.15%)	498 (2.44%)	2217 (10.86%)	2895 (14.18%)
Hepatic and hepatobiliary complications	1583 (7.76%)	492 (2.41%)	2053 (10.06%)	2811 (13.77%)
Chronic mental health disorders	789 (3.87%)	478 (2.34%)	2010 (9.85%)	2391 (11.71%)
Chronic pain	816 (4%)	464 (2.27%)	1846 (9.04%)	2296 (11.25%)
Bacteremia and sepsis	1556 (7.62%)	168 (0.82%)	545 (2.67%)	1807 (8.85%)
Stroke	829 (4.06%)	244 (1.2%)	1465 (7.18%)	1781 (8.73%)
Avascular necrosis and bone damage	869 (4.26%)	158 (0.77%)	1205 (5.9%)	1563 (7.66%)
Splenic disease	611 (2.99%)	172 (0.84%)	1067 (5.23%)	1421 (6.96%)
Acute renal failure	1086 (5.32%)	139 (0.68%)	615 (3.01%)	1405 (6.88%)
Ocular complications	21 (0.1%)	24 (0.12%)	1049 (5.14%)	1057 (5.18%)
Transfusion complications	400 (1.96%)	38 (0.19%)	688 (3.37%)	910 (4.46%)
Cognitive impairment	159 (0.78%)	34 (0.17%)	669 (3.28%)	763 (3.74%)
Hydroxyurea - thrombocytopenia	370 (1.81%)	53 (0.26%)	327 (1.6%)	592 (2.9%)
Hydroxyurea - leukopenia	164 (0.8%)	37 (0.18%)	285 (1.4%)	416 (2.04%)
Dactylitis	77 (0.38%)	55 (0.27%)	309 (1.51%)	398 (1.95%)
Priapism	126 (0.62%)	136 (0.67%)	259 (1.27%)	315 (1.54%)
Myocardial infarction	222 (1.09%)	28 (0.14%)	129 (0.63%)	294 (1.44%)
Multi-organ failure	150 (0.73%)	8 (0.04%)	24 (0.12%)	170 (0.83%)
Leg ulcers	37 (0.18%)	14 (0.07%)	102 (0.5%)	126 (0.62%)
Hydroxyurea—oligospermia/azospermia	0 (0%)	0 (0%)	19 (0.09%)	19 (0.09%)

*Combined = Inpatient (any position) or ER or ambulatory

**Table 4:** Prevalence of SCD comorbidities by age group (no enrollment requirement).

Prevalence by Age Category**				
	N (%)			
Comorbidities*	<18	18–45	46–64	Total N
**Total**	**7,921**	**10,869**	**4,659**	**20,411**
Vaso-occlusive pain	4561 (57.58%)	5936 (54.61%)	1829 (39.26%)	11478 (56.23%)
Infections (non-specific)	4336 (54.74%)	5333 (49.07%)	2363 (50.72%)	11485 (56.27%)
Fever	3827 (48.31%)	2694 (24.79%)	856 (18.37%)	7163 (35.09%)
Acute chest syndrome	2240 (28.28%)	2693 (24.78%)	966 (20.73%)	5700 (27.93%)
Cardiovascular including pulmonary hypertension	731 (9.23%)	2893 (26.62%)	2096 (44.99%)	5527 (27.08%)
Fatigue	485 (6.12%)	2473 (22.75%)	1656 (35.54%)	4462 (21.86%)
Asthma	2015 (25.44%)	1325 (12.19%)	666 (14.29%)	3778 (18.51%)
Chronic lung disease	1175 (14.83%)	1539 (14.16%)	1069 (22.94%)	3679 (18.02%)
Sleep disordered breathing and nocturnal hypoxemia	935 (11.8%)	1252 (11.52%)	763 (16.38%)	2854 (13.98%)
Chronic renal disease	332 (4.19%)	1302 (11.98%)	1265 (27.15%)	2806 (13.75%)
Hepatic and hepatobiliary complications	667 (8.42%)	1367 (12.58%)	719 (15.43%)	2700 (13.23%)
Chronic mental health disorders	286 (3.61%)	1364 (12.55%)	742 (15.93%)	2307 (11.3%)
Chronic pain	212 (2.68%)	1379 (12.69%)	729 (15.65%)	2237 (10.96%)
Bacteremia and sepsis	433 (5.47%)	929 (8.55%)	434 (9.32%)	1776 (8.7%)
Stroke	467 (5.9%)	630 (5.8%)	702 (15.07%)	1742 (8.53%)
Avascular necrosis and bone damage	245 (3.09%)	992 (9.13%)	386 (8.29%)	1535 (7.52%)
Splenic disease	685 (8.65%)	505 (4.65%)	239 (5.13%)	1397 (6.84%)
Acute renal failure	116 (1.46%)	572 (5.26%)	700 (15.02%)	1366 (6.69%)
Ocular complications	145 (1.83%)	533 (4.9%)	377 (8.09%)	1018 (4.99%)
Transfusion complications	361 (4.56%)	444 (4.09%)	125 (2.68%)	890 (4.36%)
Cognitive impairment	358 (4.52%)	178 (1.64%)	189 (4.06%)	713 (3.49%)
Hydroxyurea - thrombocytopenia	161 (2.03%)	257 (2.36%)	162 (3.48%)	575 (2.82%)
Hydroxyurea - leukopenia	142 (1.79%)	134 (1.23%)	134 (2.88%)	405 (1.98%)
Dactylitis	105 (1.33%)	163 (1.5%)	122 (2.62%)	390 (1.91%)
Priapism	97 (1.22%)	213 (1.96%)	19 (0.41%)	312 (1.53%)
Myocardial infarction	4 (0.05%)	92 (0.85%)	194 (4.16%)	286 (1.4%)
Multi-organ failure	21 (0.27%)	77 (0.71%)	70 (1.5%)	169 (0.83%)
Leg ulcers	2 (0.03%)	65 (0.6%)	49 (1.05%)	115 (0.56%)
Hydroxyurea—oligospermia/azospermia	0 (0%)	17 (0.16%)	3 (0.06%)	19 (0.09%)

*Combined = Inpatient (any position) or ER or ambulatory

**Prevalence in each age window. Each patient may be in multiple age windows

In the primary analysis, vaso-occlusive pain (59%) was the most prevalent condition for persons age <18, followed by infections (non-specific) (57%), fever (50%), and acute chest syndrome (29%) (Table 1). In the 18–45 age group, vaso-occlusive pain was also the most prevalent condition (56%), followed by infections (non-specific) (51%) and cardiovascular including pulmonary hypertension (28%). In the 46–64 age group, infections (non-specific) was the most prevalent (52%), followed by cardiovascular including pulmonary hypertension (46%) and vaso-occlusive pain (40%). Using the <18 group as a reference point, the prevalence of vaso-occlusive pain, fever, and acute chest syndrome declined in older populations.

Considering place of service, vaso-occlusive pain accounted for the most inpatient services (39%), followed by infections (24%) and acute chest syndrome (22%) (Table 3). Across all places of service, infections (non-specific), vaso-occlusive pain, and fever were the most common comorbidities.

Removing the 12-month enrollment requirement had a relatively modest reduction on the prevalence estimates (Table 4). The greatest change, expressed as an absolute difference in percentage, was infections in the 18–45 group, with a difference that was less than 3 percentage points.

The prevalence of complications was generally higher across age groups for males versus females, with the exception of infections, fatigue, and chronic mental health disorders (S1a and S1b Tables in S1 File). Differences between males and females ranged from <1% (most complications) to 17% [vaso-occlusive pain, ages 18–45, males > females; infections (non-specific), ages 18–45, females > males]

Table 5 shows total comorbidity burden as estimated by examining records across service units. Across all service categories, about 8% of patients had no recorded comorbidities; 44% had between one and three comorbidities; 29% had between 4 and 6 comorbidities, and 19% had 7 or more recorded comorbidities.

**Table 5:** Number of comorbidities (no enrollment requirement).

Prevalence by Service Category				
Number of morbidities	Inpatient services	ER services	Ambulatory services	Combined*
0	9644 (47.25%)	9266 (45.4%)	2603 (12.75%)	1704 (8.35%)
1	2174 (10.65%)	5227 (25.61%)	3945 (19.33%)	3025 (14.82%)
2	2032 (9.96%)	2958 (14.49%)	3886 (19.04%)	3181 (15.58%)
3	1739 (8.52%)	1646 (8.06%)	3148 (15.42%)	2846 (13.94%)
4	1372 (6.72%)	759 (3.72%)	2286 (11.20%)	2430 (11.91%)
5	1012 (4.96%)	337 (1.65%)	1651 (8.09%)	1883 (9.23%)
6	827 (4.05%)	128 (0.63%)	1123 (5.50%)	1554 (7.61%)
7	538 (2.64%)	50 (0.24%)	745 (3.65%)	1103 (5.40%)
8	360 (1.76%)	26 (0.13%)	439 (2.15%)	844 (4.14%)
9	253 (1.24%)	6 (0.03%)	267 (1.31%)	594 (2.91%)
10	158 (0.77%)	6 (0.03%)	121 (0.59%)	419 (2.05%)
11	112 (0.55%)	1 (0.00%)	97 (0.48%)	271 (1.33%)
12+	190 (0.93%)	1 (0.00%)	100 (0.49%)	557 (2.73%)

*Combined = Inpatient (any position) or ER or ambulatory

## Discussion

Using a national database of commercial insurance plans, we sought to characterize the prevalence of comorbidities associated with sickle cell disease. We found very high prevalence rates for conditions that are characteristic of this condition, particularly in persons under age 18. For older age groups, comorbidities that are common in non-SCD patients, but made more common or more severe by SCD, increased substantially to levels that are significantly higher than those seen in persons without the disease. For example, in the National Health Interview Survey, less than 3% of persons ages 46–64 report having a stroke, compared to nearly 18% in this sample [13]. The excess burden of complications for persons with SCD is similarly great for many other comorbidities, including cardiovascular disease, fatigue, and asthma.

To our knowledge this is the first comprehensive accounting of the prevalence of comorbidities that are characteristic or common to persons with SCD in persons under age 65. These data may be of interest to persons who seek to understand the burden of disease for SCD among insured persons and their dependents. Such information may be useful for those who focus on interventions focused on treating SCD and its sequelae. For those evaluating curative therapies such as novel gene therapies or bone marrow transplant, our estimates may provide useful guidance to understand how the health burden of illness can be impacted. This work calls attention to the potential cost-benefit of preventative and screening services for SCD.

There is a paucity of current sickle cell registries or databases with which to compare our results. While several databases are currently being promoted and populated, the most comprehensive natural history and prevalence data comes from the Cooperative Study of Sickle Cell Disease (CSSCD), established in 1977, and other studies focused on individual conditions [14–24]. Despite the differing methodologies several themes are consistent. As described previously, we find that acute complications that are commonly thought of as being associated with sickle cell such as pain, acute chest syndrome and infectious concerns have a high prevalence [15, 19, 22, 24]. Consistent with what has been reported, we find that chronic complications that are not specific to SCD, but are much more common in persons with the condition, such as chronic pain, cardio-vascular conditions including pulmonary hypertension and renal disease are more prevalent at older ages in SCD patients [19, 21, 25]. Similar to what is seen in persons without SCD, the prevalence of asthma is common in all age groups [21, 26]. Importantly, our data call attention to the high prevalence of several complications which were historically not considered as common issues for SCD patients, but have later been confirmed in studies. The impact of fatigue on quality of life is increasingly acknowledged, and we find claims related to fatigue are more common than those for vaso-occlusive pain in older adults with sickle cell [27]. Similarly, there is more awareness of the impact of sickle cell on mental health and we find more claims for these conditions than for chronic pain in all age groups [28, 29]. We find significantly fewer than expected claims for some complications; for example, avascular necrosis (AVN), leg ulcers and cognitive impairment. While our analysis does not allow for a definitive explanation, it may be that coding errors (e.g., AVN and leg ulcers being coded solely as pain), or lack of awareness (e.g., cognitive impairment) may be in part responsible.

Restricting the sample to persons enrolled for at least 12 months prior to the first recorded diagnosis had little impact on the prevalence estimates. While such restrictions are common in studies using administrative claims to provide enough observation time for the condition to be recorded, these studies are typically among persons with illness where comorbidity is otherwise less prevalent. Multiple comorbidities are far more common among persons with SCD, particularly as they age. As such, restrictions may not be needed for prevalence estimates, since they tend to reduce the available sample.

We note several limitations to our study. First, the sample is restricted to persons under age 65 who are enrolled in commercial insurance plans and their dependents. This population is not representative of all SCD patients, since the latter would include those enrolled in Medicaid or Medicare, either due to low-income status, age, or disability. Based on CMS estimates for the number of prevalent SCD cases, we believe that our study represents approximately one-third of the population of individuals with SCD in the United States [30]. Persons in these populations may have higher rates of comorbidities than what is reported in this sample. Second, administrative codes can be unreliable for some of the complications listed here. Our algorithm was designed to remove “rule-out” codes; that is, those that might appear once during an evaluation where the condition of interest is not confirmed. In addition, administrative codes have been shown to be manipulated or prioritized to maximize reimbursement in certain situations. In addition, some less specific codes that are common but underprioritized by healthcare providers (e.g., fatigue) are likely underreported here. Some coding might be inaccurate because of provider error. For example, dactylitis is rare among persons over age two, but appears to increase in prevalence in this sample. It is possible that particular symptoms that are common in SCD patients at older ages (e.g., foot pain) are being misinterpreted as conditions specific to dactylitis. Some codes are non-specific and could apply to multiple conditions (e.g., fever and infection). Other codes may be overlooked, either as providers do not think to report them, or conditions require special evaluations not performed routinely (e.g. thrombocytopenia or azospermia associated with hydroxyurea). Additional conditions may be underrepresented as providers may not ask, and patients might not report them (e.g. priapism). Our focus was on the most commonly recorded conditions, and those that are characteristic of SCD. Some conditions that are known to be common in older age groups may be underreported simply because SCD patients are less likely to seek care for them as they age. For example, it is known that older SCD patients learn to avoid ED or hospital visits for vaso-occlusive pain by self-management at home or simply because they find the experience of those services to be worse than the condition itself [31].

The substantial mortality of SCD will reduce the number of available persons in any given year. The MarketScan database does not provide death status after 2016, and thus we cannot estimate the number alive for the total population.

It is possible that the prevalence estimates among persons age <18 are reasonable representations of the population of SCD persons in that age category. The great majority of these individuals are dependents of employed adults, and thus with greater access to healthcare than the children of adults with no insurance, underinsurance, or enrolled in Medicaid.

## Conclusions

In summary, we have estimated the claims-based prevalence of multiple comorbidities that are known to be specific for or associated with SCD. These estimates may be useful for epidemiologists, insurers, those conducting budget impact analyses or burden of illness studies, and for conducting cost-effectiveness analyses of new and existing technologies aimed at treatment of SCD. The nature of sampling—persons with commercial insurance through an employer and their dependents—should be taken into account when comparing these results to other populations.

## Supporting information

S1 File (DOCX)
