# Correction: Prevalence of comorbidities associated with sickle cell disease among non-elderly individuals with commercial insurance–A retrospective cohort study

**DOI:** 10.1371/journal.pone.0328920

**Published:** 2025-07-22

**Authors:** Scott D. Ramsey, M. A. Bender, Li Li, Kate M. Johnson, Boshen Jiao, Beth Devine, Anirban Basu

S1 Appendix A is omitted from the list of Supporting Information. It can be viewed below.

## S1 Appendix A: ICD Codes

**Table:** 

Condition	ICD9	ICD10	Identification
**Acute anemia**	079.83 Parvovirus B19 284.12- Other drug-induced pancytopenia 285.3- Antineoplastic chemotherapy induced anemia	B34.3 Parvovirus infection, unspecified D57.212- Sickle-cell/Hb-C disease with splenic sequestration D57.412- Sickle-cell thalassemia with splenic sequestration D57.812- Other sickle-cell disorders with splenic sequestration D57.819- Other sickle-cell disorders with crisis, unspecified D60.1- Transient acquired pure red cell aplasia D61.810- Antineoplastic chemotherapy induced pancytopenia D61.811- Other drug-induced pancytopenia D64.81- Anemia due to antineoplastic chemotherapy	At least 1 inpatient, SNF, HHA, HOP or Carrier claim with DX code within 12 months
**Liver disease**	571.9-Unspecified chronic liver disease w/o mention of alcohol 573.9-Unspecified disorder of liver	K75.89-Other specified inflammatory liver diseases K75.9-Inflammatory liver disease, unspecified K76.89-Other specified diseases of liver K76.9-Liver disease, unspecified K77-Liver disorders in diseases classified elsewhere	
**Hepatobiliary Complications –** **Cholecystitis and Gall Bladder Disease**	560.31- Gallstone ileus 570- Acute and subacute hepatic failure without coma 571.6-Biliary cirrhosis 571.9-Unspecified chronic liver disease without mention of alcohol 572.2- Hepatic encephalopathy 572.3-Portal hypertension 572.4-Hepatorenal syndrome 574.00 Calculus of gallbladder with acute cholecystitis, without mention of obstruction 574.01 Calculus of gallbladder with acute cholecystitis, with obstruction 574.10 Calculus of gallbladder with other cholecystitis, without mention of obstruction 574.11 Calculus of gallbladder with other cholecystitis, with obstruction 574.20 Calculus of gallbladder without mention of cholecystitis, without mention of obstruction 574.21 Calculus of gallbladder without mention of cholecystitis, with obstruction 574.30 Calculus of bile duct with acute cholecystitis, without mention of obstruction 574.31 Calculus of bile duct with acute cholecystitis, with obstruction 574.40 Calculus of bile duct with other cholecystitis, without mention of obstruction 574.41 Calculus of bile duct with other cholecystitis, with obstruction 574.50 Calculus of bile duct without mention of cholecystitis, without mention of obstruction 574.51 Calculus of bile duct without mention of cholecystitis, with obstruction 574.60 Calculus of gallbladder and bile duct with acute	K56.3-Gallstone ileus K72.00 Acute and subacute hepatic failure without coma K81.0-Acute cholecystitis K81.1-Chronic cholecystitis K81.2-Acute cholecystitis with chronic cholecystitis K81.9-Cholecystitis, unspecified K80.00-Calculus of gallbladder with acute cholecystitis without obstruction K80.01-Calculus of gallbladder with acute cholecystitis with obstruction K80.10-Calculus of gallbladder with chronic cholecystitis without obstruction K80.11-Calculus of gallbladder with chronic cholecystitis with obstruction K80.12-Calculus of gallbladder with acute and chronic cholecystitis without obstruction K80.13-Calculus of gallbladder with acute and chronic cholecystitis with obstruction K80.18-Calculus of gallbladder with other cholecystitis without obstruction K80.19-Calculus of gallbladder with other cholecystitis with obstruction K80.20-Calculus of gallbladder without cholecystitis without obstruction K80.21-Calculus of gallbladder without cholecystitis with obstruction K80.30 Calculus of bile duct with cholangitis unspecified, without obstruction K80.31 Calculus of bile duct with cholangitis unspecified, with obstruction K80.32 Calculus of bile duct with acute cholangitis without obstruction K80.33 Calculus of bile duct with acute cholangitis with obstruction K80.34 Calculus of bile duct with chronic cholangitis without obstruction K80.35 Calculus of bile duct with chronic cholangitis with obstruction K80.36 Calculus of bile duct with acute and chronic cholangitis without K80.37 Calculus of bile duct with acute and chronic cholangitis with obstruction K80.40 Calculus of bile duct with cholecystitis unspecified, without obstruction K80.41 Calculus of bile duct with cholecystitis unspecified, with obstruction K80.42 Calculus of bile duct with acute cholecystitis without obstruction K80.43 Calculus of bile duct with acute cholecystitis with obstruction	
**Hepatobiliary Complications –** **Cholecystitis and Gall Bladder Disease**	cholecystitis, without mention of obstruction 574.61 Calculus of gallbladder and bile duct with acute cholecystitis, with obstruction 574.70 Calculus of gallbladder and bile duct with other cholecystitis, without mention of obstruction 574.71 Calculus of gallbladder and bile duct with other cholecystitis, with obstruction 574.80 Calculus of gallbladder and bile duct with acute and chronic cholecystitis, without mention of obstruction 574.81 Calculus of gallbladder and bile duct with acute and chronic cholecystitis, with obstruction 574.90 Calculus of gallbladder and bile duct without cholecystitis, without mention of obstruction 574.91 Calculus of gallbladder and bile duct without cholecystitis, with obstruction 575.0- Acute cholecystitis 575.1- Other cholecystitis 575.3-Hydrops of gallbladder 575.10- Cholecystitis, unspecified 575.11- Chronic cholecystitis 575.12- Acute and chronic cholecystitis 575.2- Obstruction of gallbladder 575.4- Perforation of gallbladder 575.6- Cholesterolosis of gallbladder 576.1- Cholangitis 576.2- Obstruction of bile duct 576.3- Perforation of bile duct 576.4- Fistula of bile duct 576.5- Spasm of sphincter of Oddi 576.8- Other specified disorders of biliary tract	K80.44 Calculus of bile duct with chronic cholecystitis without obstruction K80.45 Calculus of bile duct with chronic cholecystitis with obstruction K80.46 Calculus of bile duct with acute and chronic cholecystitis without obstruction K80.47 Calculus of bile duct with acute and chronic cholecystitis with obstruction K80.50 Calculus of bile duct without cholangitis or cholecystitis without obstruction K80.51 Calculus of bile duct without cholangitis or cholecystitis with obstruction K80.60 Calculus of gallbladder and bile duct with cholecystitis unspecified, without obstruction K80.61 Calculus of gallbladder and bile duct with cholecystitis unspecified, with obstruction K80.62 Calculus of gallbladder and bile duct with acute cholecystitis without obstruction K80.63 Calculus of gallbladder and bile duct with acute cholecystitis with obstruction K80.64 Calculus of gallbladder and bile duct with chronic cholecystitis without obstruction K80.65 Calculus of gallbladder and bile duct with chronic cholecystitis with obstruction K80.66 Calculus of gallbladder and bile duct with acute and chronic cholecystitis without obstruction K80.67 Calculus of gallbladder and bile duct with acute and chronic cholecystitis with obstruction K80.70 Calculus of gallbladder and bile duct without cholecystitis without obstruction K80.71 Calculus of gallbladder and bile duct without cholecystitis with obstruction K80.80 Other cholelithiasis without obstruction K80.81 Other cholelithiasis with obstruction K82.0-Obstruction of gallbladder K82.1-Hydrops of gallbladder K82.2-Perforation of gallbladder K82.8-Other specified diseases of gallbladder K82.9-Disease of gallbladder, unspecified K82.A1-Gangrene of gallbladder in cholecystitis K82.A2-Perforation of gallbladder in cholecystitis R93.2-Abnormal findings on diagnostic imaging of liver and biliary tract K72.00-Acute and subacute hepatic failure without coma K72.01-Acute and subacute hepatic failure with coma K74.0-Hepatic fibrosis K74.1-Hepatic sclerosis K74.2-Hepatic fibrosis with hepatic sclerosis	
**Myocardial infarction**	410-Myocardial infarction 410.9-Myocardial infarction of unspecified site 411.81-Acute coronary occlusion w/o myocardial infarction 410.01-Acute myocardial infarction of anterolateral wall, initial episode of care 410.11-Acute myocardial infarction of other anterior wall, initial episode of care 410.21-Acute myocardial infarction of inferolateral wall, initial episode of care 410.31-Acute myocardial infarction of inferoposterior wall, initial episode of care 410.41-Acute myocardial infarction of other inferior wall, initial episode of care 410.51-Acute myocardial infarction of other lateral wall, initial episode of care 410.61-True posterior wall infarction, initial episode of care 410.71-Subendocardial infarction, initial episode of care 410.81-Acute myocardial infarction of other specified sites, initial episode of care 410.91-Acute myocardial infarction of unspecified site, initial episode of care	I21.9-Acute myocardial infarction, unspecified I21.A1-Myocardial infarction type 2 I21.A9-Other myocardial infarction type I21.01- ST elevation (STEMI) myocardial infarction involving left main coronary artery I21.02- ST elevation (STEMI) myocardial infarction involving left anterior descending coronary artery I21.09- ST elevation (STEMI) myocardial infarction involving other coronary artery of anterior wall I21.11- ST elevation (STEMI) myocardial infarction involving right coronary artery I21.19- ST elevation (STEMI) myocardial infarction involving other coronary artery of inferior wall I21.21- ST elevation (STEMI) myocardial infarction involving left circumflex coronary artery I21.29- ST elevation (STEMI) myocardial infarction involving other sites I21.3- ST elevation (STEMI) myocardial infarction of unspecified site I21.4- Non-ST elevation (NSTEMI) myocardial infarction I22- Subsequent ST elevation (STEMI) and non-ST elevation (NSTEMI) myocardial infarction I22.1- Subsequent ST elevation (STEMI) myocardial infarction of inferior wall I22.2- Subsequent non-ST elevation (NSTEMI) myocardial infarction I22.8- Subsequent ST elevation (STEMI) myocardial infarction of other sites I22.9- Subsequent ST elevation (STEMI) myocardial infarction of unspecified site	ONLY first or second DX on the claim At least 1 inpatients claim with Dx code in 12 months.
**Pulmonary hypertension**	416.0-Primary Pulmonary hypertension 416.8-Other chronic pulmonary heart disease. 416.9-Chronic pulmonary heart disease, unspecified.	I27.0-Primary pulmonary hypertension I27.20-Pulmonary hypertension, unspecified I27.21-Secondary pulmonary arterial hypertension I27.22-Pulmonary hypertension due to left heart disease I27.23-Pulmonary hypertension due to lung diseases and hypoxia I27.24-Chronic thromboembolic pulmonary hypertension I27.29-Other secondary pulmonary hypertension I27.81 - Cor pulmonale (chronic) (due to pulmonary hypertension) I27.9 - Pulmonary heart disease, unspecified (resulting from pulmonary hypertension)	
**Other Cardiovascular Disease**	428.21- Systolic heart failure, acute 428.22- Systolic heart failure, chronic 428.23- Systolic heart failure, acute on chronic 428.30-diastolic heart dysfunction 414.9-Ischemic heart disease 798-sudden death, cause unknown 786.05-Shortness of breath, dyspnea on exertion 425.8-Cardiomyopathy 428.1-Left heart failure 428.30-Diastolic health failure, unspecified 428.31 Acute diastolic heart failure 428.32 Chronic diastolic heart failure 428.33 Acute on chronic diastolic heart failure 428.0- Congestive heart failure, unspecified 428.1- Left heart failure 428.20- Systolic heart failure, unspecified 428.21- Acute systolic heart failure 428.22- Chronic systolic heart failure 428.30- Diastolic heart failure, unspecified 428.31- Acute diastolic heart failure 428.32- Chronic diastolic heart failure 428.33- Acute on chronic diastolic heart failure 428.40- Combined systolic and diastolic heart failure, unspecified 428.42- Chronic combined systolic and diastolic heart failure 428.43- Acute on chronic combined systolic and diastolic heart failure 428.9- Heart failure, unspecified 410.00- Acute myocardial infarction of anterolateral wall, episode of care unspecified 410.01- Acute myocardial infarction of anterolateral wall, initial episode of care 410.02- Acute myocardial infarction of anterolateral wall, subsequent episode of care 410.10- Acute myocardial infarction of other anterior wall, episode of care unspecified 410.11- Acute myocardial infarction of other anterior wall, initial episode of care 410.12- Acute myocardial infarction of other anterior wall, subsequent episode of care 410.20- Acute myocardial infarction of inferolateral wall, episode of care unspecified 410.21- Acute myocardial infarction of inferolateral wall, initial episode of care 410.22- Acute myocardial infarction of inferolateral wall, subsequent episode of care 410.30- Acute myocardial infarction of inferoposterior wall, episode of care unspecified 410.31- Acute myocardial infarction of inferoposterior wall, initial episode of care	I50.21- Systolic heart failure, acute I50.22- Systolic heart failure, chronic I50.23- Systolic heart failure, acute on chronic I50.30-diastolic heart dysfunction I25.9-Ischemic heart disease I46.1-sudden death, cause unknown R06.09- Shortness of breath, dyspnea on exertion I42.9-Cardiomyopathy, unspecified I50.1-Left ventricular failure, unspecified I50.31-Acute diastolic (congestive) heart failure I50.32-Chronic diastolic (congestive) heart failure I50.33-Acute on chronic diastolic (congestive) heart failure I50.30-Unspecified diastolic (congestive) heart failure I13.0- Hypertensive heart and chronic kidney disease with heart failure and stage 1 through stage 4 chronic kidney disease, or unspecified chronic kidney disease !13.2- Hypertensive heart and chronic kidney disease with heart failure and with stage 5 chronic kidney disease, or end stage renal disease !50.1- Left ventricular failure I50.20- Unspecified systolic (congestive) heart failure I50.21- Acute systolic (congestive) heart failure I50.22- Chronic systolic (congestive) heart failure I50.23- Acute on chronic systolic (congestive) heart failure I50.30- Unspecified diastolic (congestive) heart failure I50.31- Acute diastolic (congestive) heart failure I50.32- Chronic diastolic (congestive) heart failure I50.33- Acute on chronic diastolic (congestive) heart failure I50.40- Unspecified combined systolic (congestive) and diastolic (congestive) heart failure I50.41- Acute combined systolic (congestive) and diastolic (congestive) heart failure I50.42- Chronic combined systolic (congestive) and diastolic (congestive) heart failure I50.43- Acute on chronic combined systolic (congestive) and diastolic (congestive) heart failure I50.9- Heart failure, unspecified I20.0- Unstable angina I20.1- Angina pectoris with documented spasm I20.8- Other forms of angina pectoris I20.9- Angina pectoris, unspecified I21.01- ST elevation (STEMI) myocardial infarction involving left main coronary artery I21.02- ST elevation (STEMI) myocardial infarction involving left anterior descending coronary artery I21.09- ST elevation (STEMI) myocardial infarction involving other coronary artery of anterior wall I21.11- ST elevation (STEMI) myocardial infarction involving right coronary artery	At least 1 inpatient, HOP or Carrier claim with DX code within 24 months
	410.32- Acute myocardial infarction of inferoposterior wall, subsequent episode of care 410.40- Acute myocardial infarction of other inferior wall, episode of care unspecified 410.41- Acute myocardial infarction of other inferior wall, initial episode of care 410.42- Acute myocardial infarction of other inferior wall, subsequent episode of care 410.50- Acute myocardial infarction of other lateral wall, episode of care unspecified 410.51- Acute myocardial infarction of other lateral wall, initial episode of care 410.52- Acute myocardial infarction of other lateral wall, subsequent episode of care 410.60- True posterior wall infarction, episode of care unspecified 410.61- True posterior wall infarction, initial episode of care 410.62- True posterior wall infarction, subsequent episode of care 410.70- Subendocardial infarction, episode of care unspecified 410.71- Subendocardial infarction, initial episode of care 410.72- Subendocardial infarction, subsequent episode of care 410.80- Acute myocardial infarction of other specified sites, episode of care unspecified 410.81- Acute myocardial infarction of other specified sites, initial episode of care 410.82- Acute myocardial infarction of other specified sites, subsequent episode of care 410.90- Acute myocardial infarction of unspecified site, episode of care unspecified 410.91- Acute myocardial infarction of unspecified site, initial episode of care 410.92- Acute myocardial infarction of unspecified site, subsequent episode of care 411.0- Postmyocardial infarction syndrome 411.1- Intermediate coronary syndrome 411.81- Acute coronary occlusion without myocardial infarction 411.89- Other acute and subacute forms of ischemic heart disease, other 412- Old myocardial infarction 413.0- Angina decubitus 413.1- Prinzmetal angina 413.9- Other and unspecified angina pectoris 414.00- Coronary atherosclerosis of unspecified type of vessel, native or graft 414.01- Coronary atherosclerosis of native coronary artery 414.2- Chronic total occlusion of coronary artery 414.3- Coronary atherosclerosis due to lipid rich plaque 414.4- Coronary atherosclerosis due to calcified coronary lesion 414.8- Other specified forms of chronic ischemic heart disease 414.9- Chronic ischemic heart disease, unspecified	I21.19- ST elevation (STEMI) myocardial infarction involving other coronary artery of inferior wall I21.21- ST elevation (STEMI) myocardial infarction involving left circumflex coronary artery I21.29- ST elevation (STEMI) myocardial infarction involving other sites I21.3- ST elevation (STEMI) myocardial infarction of unspecified site I21.4- Non-ST elevation (NSTEMI) myocardial infarction I22.0- Subsequent ST elevation (STEMI) myocardial infarction of anterior wall I22.1- Subsequent ST elevation (STEMI) myocardial infarction of inferior wall I22.2- Subsequent non-ST elevation (NSTEMI) myocardial infarction I22.8- Subsequent ST elevation (STEMI) myocardial infarction of other sites I2.9- Subsequent ST elevation (STEMI) myocardial infarction of unspecified site I23.6- Thrombosis of atrium, auricular appendage, and ventricle as current complications following acute myocardial infarction I23.7- Postinfarction angina I23.8- Other current complications following acute myocardial infarction I24.0- Acute coronary thrombosis not resulting in myocardial infarction I24.1- Dressler’s syndrome I24.8- Other forms of acute ischemic heart disease I24.9- Acute ischemic heart disease, unspecified I25.10- Atherosclerotic heart disease of native coronary artery without angina pectoris I25.110- Atherosclerotic heart disease of native coronary artery with unstable angina pectoris I25.111- Atherosclerotic heart disease of native coronary artery with angina pectoris with documented spasm I25.118- Atherosclerotic heart disease of native coronary artery with other forms of angina pectoris I25.119- Atherosclerotic heart disease of native coronary artery with unspecified angina pectoris I25.2- Old myocardial infarction I25.5- Ischemic cardiomyopathy I25.6- Silent myocardial ischemia I25.82- Chronic total occlusion of coronary artery I25.83- Coronary atherosclerosis due to lipid rich plaque I25.84- Coronary atherosclerosis due to calcified coronary lesion I25.89- Coronary atherosclerosis due to calcified coronary lesion I25.9- Chronic ischemic heart disease, unspecified	
**Vaso-occlusive pain episodes**	282.42-Sickle-cell thalassemia with crisis 282.64-vaso-occlusive pain 282.62- sickle cell “crisis”, NOS 282.69 Other sickle-cell disease with crisis	D57.00- Hb-SS disease with crisis, unspecified D57.219- Sickle-cell/Hb-C disease with crisis, unspecified D57.419 – Sickle-cell thalassemia with crisis D57.819 – Other sickle-cell disorders with crisis	
**Sickle Cell Crisis**	282.62 Hb-SS disease with crisis 282.64 Sickle-cell/HB-C disease with crisis 282.69 Other sickle-cell disease with crisis	D57.0 Hb-SS disease with crisis D57.00 Hb-SS disease with crisis, unspecified D57.21 Sickle-cell/Hb-C disease with crisis D57.219 Sickle-cell/Hb-C disease with crisis, unspecified D57.41 Sickle-cell thalassemia with crisis D57.419 Sickle-cell thalassemia with crisis, unspecified D57.81 Other sickle-cell disorders with crisis D57.819 Other sickle-cell disorders with crisis, unspecified	
**Gene therapy**	996.85-Complications of transplanted bone marrow V42.81 Bone marrow replaced by transplant V42.82- Peripheral stem cells replaced by transplant 733.00 Osteoporosis, unspecified 275.02 Hemochromatosis due to repeated red blood cell transfusions 296.20 Major depressive affective disorder, single episode, unspecified 296.30 Major depressive affective disorder, recurrent episode, unspecified 300.00 Anxiety state, unspecified 491.8-Other chronic bronchitis 238.77 Post-transplant lymphoproliferative disorder (PTLD) 348.39 Other encephalopathy	T86.5- Complications of stem cell transplant Z94.81- Bone marrow transplant status Z94.84-Stem cells transplant status T86.00- Unspecified complication of bone marrow transplant T86.01- Bone marrow transplant rejection T86.02- Bone marrow transplant failure T86.03- Bone marrow transplant infection T86.09- Other complications of bone marrow transplant D89.9-Disorder involving the immune mechanism, unspecified T86.00-Unspecified complication of bone marrow transplant T86.09-Other complications of bone marrow transplant M81.8- Other osteoporosis without current pathological fracture E83.111-Hemochromatosis due to repeated red blood cell transfusions F32.9-Major depressive disorder, single episode, unspecified F33.9-Major depressive disorder, recurrent, unspecified F41.9-Anxiety disorder, unspecified J84.02 Pulmonary alveolar microlithiasis J84.03 Idiopathic pulmonary hemosiderosis J84.09 Other alveolar and parieto-alveolar conditions D47.Z1-Post-transplant lymphoproliferative disorder (PTLD) I67.83 Posterior reversible encephalopathy syndrome	
**Gene therapy – (induced mutagenesis)**	198.0 Secondary malignant neoplasm of kidney 198.1 Secondary malignant neoplasm of other urinary organs 198.2 Secondary malignant neoplasm of skin 198.3 Secondary malignant neoplasm of brain and spinal cord 198.4 Secondary malignant neoplasm of other parts of nervous system 198.5 Secondary malignant neoplasm of bone and bone marrow 198.6 Secondary malignant neoplasm of ovary 198.7 Secondary malignant neoplasm of adrenal gland 198.81 Secondary malignant neoplasm of breast 198.82 Secondary malignant neoplasm of genital organs 198.89 Secondary malignant neoplasm of other specified sites	C79.00-Secondary malignant neoplasm of unspecified kidney and renal pelvis C79.01-Secondary malignant neoplasm of right kidney and renal pelvis C79.02-Secondary malignant neoplasm of left kidney and renal pelvis C79.10-Secondary malignant neoplasm of unspecified urinary organs C79.11-Secondary malignant neoplasm of bladder C79.19-Secondary malignant neoplasm of other urinary organs C79.2-Secondary malignant neoplasm of skin C79.31-Secondary malignant neoplasm of brain C79.32-Secondary malignant neoplasm of cerebral meninges C79.40-Secondary malignant neoplasm of unspecified part of nervous system C79.49-Secondary malignant neoplasm of other parts of nervous system C79.51-Secondary malignant neoplasm of bone C79.52-Secondary malignant neoplasm of bone marrow C79.60-Secondary malignant neoplasm of unspecified ovary C79.61-Secondary malignant neoplasm of right ovary C79.62-Secondary malignant neoplasm of left ovary C79.70-Secondary malignant neoplasm of unspecified adrenal gland C79.71-Secondary malignant neoplasm of right adrenal gland C79.72-Secondary malignant neoplasm of left adrenal gland C79.81-Secondary malignant neoplasm of breast C79.82-Secondary malignant neoplasm of genital organs C79.89-Secondary malignant neoplasm of other specified sites C79.9-Secondary malignant neoplasm of unspecified site	
**Transplant Complications**	996.85-Complications of transplanted bone marrow V42.81 Bone marrow replaced by transplant V42.82- Peripheral stem cells replaced by transplant 279.50 Graft-versus-host disease, unspecified 279.51 Acute graft-versus-host disease 279.52 Chronic graft-versus-host disease 279.53 Acute on chronic graft-versus-host disease 733.00 Osteoporosis, unspecified 275.02 Hemochromatosis due to repeated red blood cell transfusions 296.20 Major depressive affective disorder, single episode, unspecified 296.30 Major depressive affective disorder, recurrent episode, unspecified 300.00 Anxiety state, unspecified 491.8-Other chronic bronchitis 238.77 Post-transplant lymphoproliferative disorder (PTLD) 348.39 Other encephalopathy	T86.5-Complications of stem cell transplant Z94.81- Bone marrow transplant status Z94.84-Stem cells transplant status T86.00-Unspecified complication of bone marrow transplant T86.01-Bone marrow transplant rejection T86.02-Bone marrow transplant failure T86.03-Bone marrow transplant infection T86.09-Other complications of bone marrow transplant D89.9-Disorder involving the immune mechanism, unspecified T86.00-Unspecified complication of bone marrow transplant T86.09-Other complications of bone marrow transplant D89.81 Graft-versus-host disease D89.810 Acute graft-versus-host disease D89.811 Chronic graft-versus-host disease D89.812 Acute on chronic graft-versus-host disease D89.813 …… unspecified M81.8- Other osteoporosis without current pathological fracture E83.111-Hemochromatosis due to repeated red blood cell transfusions F32.9-Major depressive disorder, single episode, unspecified F33.9-Major depressive disorder, recurrent, unspecified F41.9-Anxiety disorder, unspecified J84.02 Pulmonary alveolar microlithiasis J84.03 Idiopathic pulmonary hemosiderosis J84.09 Other alveolar and parieto-alveolar conditions D47.Z1 Post-transplant lymphoproliferative disorder (PTLD) I67.83 Posterior reversible encephalopathy syndrome	
**Transplant– (induced mutagenesis)**	198.0 Secondary malignant neoplasm of kidney 198.1 Secondary malignant neoplasm of other urinary organs 198.2 Secondary malignant neoplasm of skin 198.3 Secondary malignant neoplasm of brain and spinal cord 198.4 Secondary malignant neoplasm of other parts of nervous system 198.5 Secondary malignant neoplasm of bone and bone marrow 198.6 Secondary malignant neoplasm of ovary 198.7 Secondary malignant neoplasm of adrenal gland 198.81 Secondary malignant neoplasm of breast 198.82 Secondary malignant neoplasm of genital organs 198.89 Secondary malignant neoplasm of other specified sites	C79.00-Secondary malignant neoplasm of unspecified kidney and renal pelvis C79.01-Secondary malignant neoplasm of right kidney and renal pelvis C79.02-Secondary malignant neoplasm of left kidney and renal pelvis C79.10-Secondary malignant neoplasm of unspecified urinary organs C79.11-Secondary malignant neoplasm of bladder C79.19-Secondary malignant neoplasm of other urinary organs C79.2-Secondary malignant neoplasm of skin C79.31-Secondary malignant neoplasm of brain C79.32-Secondary malignant neoplasm of cerebral meninges C79.40-Secondary malignant neoplasm of unspecified part of nervous system C79.49-Secondary malignant neoplasm of other parts of nervous system C79.51-Secondary malignant neoplasm of bone C79.52-Secondary malignant neoplasm of bone marrow C79.60-Secondary malignant neoplasm of unspecified ovary C79.61-Secondary malignant neoplasm of right ovary C79.62-Secondary malignant neoplasm of left ovary C79.70-Secondary malignant neoplasm of unspecified adrenal gland C79.71-Secondary malignant neoplasm of right adrenal gland C79.72-Secondary malignant neoplasm of left adrenal gland C79.81-Secondary malignant neoplasm of breast C79.82-Secondary malignant neoplasm of genital organs C79.89-Secondary malignant neoplasm of other specified sites C79.9-Secondary malignant neoplasm of unspecified site	
