# Supplementary material for: Prevalence of comorbidities associated with sickle cell disease among non-elderly individuals with commercial insurance–A retrospective cohort study
Source: PLoS One. 2022 Nov 29;17(11):e0278137. doi: 10.1371/journal.pone.0278137 (PMC9707783; doi:10.1371/journal.pone.0278137)
Supplement: S1 File — (DOCX) [file pone.0278137.s001.docx]

**Supplement Table 1a: Prevalence of SCD Comorbidities by Age Group (12-month enrollment requirement), Male**

| **Prevalence by Age Category**** | | | | |
| --- | --- | --- | --- | --- |
|  | **N (%)** | | |  |
| **Comorbidities*** | **<18** | **18-45** | **46-64** | **Total N** |
| **Total** | **3,750** | **4,028** | **1,666** | **8,632** |
| Vaso-occlusive pain | 2247 (59.92%) | 2539 (63.03%) | 665 (39.92%) | 5177 (59.97%) |
| Infections (non-specific) | 2074 (55.31%) | 1664 (41.31%) | 768 (46.1%) | 4369 (50.61%) |
| Fever | 1892 (50.45%) | 1167 (28.97%) | 312 (18.73%) | 3296 (38.18%) |
| Acute chest syndrome | 1149 (30.64%) | 1223 (30.36%) | 392 (23.53%) | 2691 (31.17%) |
| Cardiovascular including pulmonary hypertension | 367 (9.79%) | 1143 (28.38%) | 804 (48.26%) | 2255 (26.12%) |
| Asthma | 1077 (28.72%) | 448 (11.12%) | 177 (10.62%) | 1628 (18.86%) |
| Chronic lung disease | 608 (16.21%) | 674 (16.73%) | 397 (23.83%) | 1645 (19.06%) |
| Fatigue | 211 (5.63%) | 755 (18.74%) | 546 (32.77%) | 1485 (17.20%) |
| Sleep disordered breathing and nocturnal hypoxemia | 467 (12.45%) | 597 (14.82%) | 312 (18.73%) | 1337 (15.49%) |
| Chronic renal disease | 163 (4.35%) | 534 (13.26%) | 525 (31.51%) | 1187 (13.75%) |
| Hepatic and hepatobiliary complications | 329 (8.77%) | 595 (14.77%) | 281 (16.87%) | 1184 (13.72%) |
| Chronic pain | 88 (2.35%) | 537 (13.33%) | 251 (15.07%) | 849 (9.84%) |
| Bacteremia and sepsis | 229 (6.11%) | 401 (9.96%) | 184 (11.04%) | 802 (9.29%) |
| Stroke | 243 (6.48%) | 263 (6.53%) | 296 (17.77%) | 775 (8.98%) |
| Avascular necrosis and bone damage | 129 (3.44%) | 476 (11.82%) | 131 (7.86%) | 701 (8.12%) |
| Chronic mental health disorders | 125 (3.33%) | 369 (9.16%) | 198 (11.88%) | 675 (7.82%) |
| Acute renal failure | 62 (1.65%) | 308 (7.65%) | 312 (18.73%) | 669 (7.75%) |
| Splenic disease | 361 (9.63%) | 227 (5.64%) | 94 (5.64%) | 668 (7.74%) |
| Ocular complications | 85 (2.27%) | 254 (6.31%) | 140 (8.4%) | 466 (5.40%) |
| Transfusion complications | 174 (4.64%) | 185 (4.59%) | 39 (2.34%) | 390 (4.52%) |
| Cognitive impairment | 208 (5.55%) | 69 (1.71%) | 86 (5.16%) | 358 (4.15%) |
| Priapism | 95 (2.53%) | 210 (5.21%) | 18 (1.08%) | 311 (3.60%) |
| Hydroxyurea - thrombocytopenia | 89 (2.37%) | 125 (3.1%) | 73 (4.38%) | 284 (3.29%) |
| Hydroxyurea - leukopenia | 85 (2.27%) | 52 (1.29%) | 52 (3.12%) | 188 (2.18%) |
| Dactylitis | 53 (1.41%) | 56 (1.39%) | 54 (3.24%) | 163 (1.89%) |
| Myocardial infarction | 4 (0.11%) | 44 (1.09%) | 101 (6.06%) | 147 (1.70%) |
| Multi-organ failure | 10 (0.27%) | 39 (0.97%) | 27 (1.62%) | 76 (0.88%) |
| Leg ulcers | 1 (0.03%) | 46 (1.14%) | 23 (1.38%) | 70 (0.81%) |
| Hydroxyurea - oligospermia/azospermia | 0 (0%) | 17 (0.42%) | 3 (0.18%) | 19 (0.22%) |

*Combined = Inpatient (any position) or ER or ambulatory

**Prevalence in each age window. Each patient may be in multiple age windows

**Supplement Table 1b: Prevalence of SCD Comorbidities by Age Group (12-month enrollment requirement), Female**

| **Prevalence by Age Category**** | | | | |
| --- | --- | --- | --- | --- |
|  | **N (%)** | | |  |
| **Comorbidities*** | **<18** | **18-45** | **46-64** | **Total N** |
| **Total** | **3,752** | **6,155** | **2,793** | **11,574** |
| Infections (non-specific) | 2196 (58.53%) | 3567 (57.95%) | 1567 (56.1%) | 7000 (60.48%) |
| Vaso-occlusive pain | 2188 (58.32%) | 3189 (51.81%) | 1108 (39.67%) | 6133 (52.99%) |
| Fever | 1892 (50.43%) | 1468 (23.85%) | 538 (19.26%) | 3797 (32.81%) |
| Cardiovascular including pulmonary hypertension | 342 (9.12%) | 1687 (27.41%) | 1264 (45.26%) | 3200 (27.65%) |
| Fatigue | 270 (7.2%) | 1691 (27.47%) | 1098 (39.31%) | 2948 (25.47%) |
| Acute chest syndrome | 1054 (28.09%) | 1413 (22.96%) | 568 (20.34%) | 2950 (25.49%) |
| Asthma | 909 (24.23%) | 835 (13.57%) | 479 (17.15%) | 2122 (18.33%) |
| Chronic lung disease | 552 (14.71%) | 843 (13.7%) | 663 (23.74%) | 2000 (17.28%) |
| Chronic mental health disorders | 157 (4.18%) | 972 (15.79%) | 536 (19.19%) | 1610 (13.91%) |
| Chronic renal disease | 157 (4.18%) | 745 (12.1%) | 725 (25.96%) | 1591 (13.75%) |
| Sleep disordered breathing and nocturnal hypoxemia | 454 (12.1%) | 632 (10.27%) | 441 (15.79%) | 1489 (12.87%) |
| Hepatic and hepatobiliary complications | 325 (8.66%) | 755 (12.27%) | 429 (15.36%) | 1486 (12.84%) |
| Chronic pain | 119 (3.17%) | 817 (13.27%) | 472 (16.9%) | 1367 (11.81%) |
| Stroke | 219 (5.84%) | 359 (5.83%) | 400 (14.32%) | 952 (8.23%) |
| Bacteremia and sepsis | 200 (5.33%) | 514 (8.35%) | 246 (8.81%) | 954 (8.24%) |
| Avascular necrosis and bone damage | 104 (2.77%) | 493 (8.01%) | 247 (8.84%) | 816 (7.05%) |
| Splenic disease | 319 (8.5%) | 272 (4.42%) | 144 (5.16%) | 721 (6.23%) |
| Acute renal failure | 50 (1.33%) | 255 (4.14%) | 379 (13.57%) | 677 (5.85%) |
| Ocular complications | 58 (1.55%) | 268 (4.35%) | 231 (8.27%) | 541 (4.67%) |
| Transfusion complications | 182 (4.85%) | 244 (3.96%) | 84 (3.01%) | 491 (4.24%) |
| Cognitive impairment | 149 (3.97%) | 107 (1.74%) | 103 (3.69%) | 353 (3.05%) |
| Hydroxyurea - thrombocytopenia | 72 (1.92%) | 130 (2.11%) | 87 (3.11%) | 287 (2.48%) |
| Dactylitis | 52 (1.39%) | 104 (1.69%) | 67 (2.4%) | 223 (1.93%) |
| Hydroxyurea - leukopenia | 57 (1.52%) | 80 (1.3%) | 81 (2.9%) | 215 (1.86%) |
| Myocardial infarction | 0 (0%) | 47 (0.76%) | 91 (3.26%) | 137 (1.18%) |
| Multi-organ failure | 11 (0.29%) | 38 (0.62%) | 43 (1.54%) | 92 (0.79%) |
| Leg ulcers | 1 (0.03%) | 19 (0.31%) | 25 (0.9%) | 45 (0.39%) |

*Combined = Inpatient (any position) or ER or ambulatory

**Prevalence in each age window. Each patient may be in multiple age windows
